# Primary Care Practitioner Perspectives on the Role of Primary Care in Dementia Diagnosis and Care

**DOI:** 10.1001/jamanetworkopen.2023.36030

**Published:** 2023-09-28

**Authors:** Alissa Bernstein Sideman, Melissa Ma, Alma Hernandez de Jesus, Cecilia Alagappan, Na’amah Razon, Daniel Dohan, Anna Chodos, Tala Al-Rousan, Loren I. Alving, Freddi Segal-Gidan, Howie Rosen, Katherine P. Rankin, Katherine L. Possin, Soo Borson

**Affiliations:** 1 Philip R. Lee Institute for Health Policy Studies, University of California, San Francisco; 2 Department of Humanities and Social Sciences, University of California, San Francisco; 3 Department of Neurology, University of California, San Francisco; 4 Global Brain Health Institute, University of California, San Francisco; 5 Department of Family and Community Medicine, University of California, Davis, Sacramento; 6 Division of Geriatrics, Department of Medicine, University of California, San Francisco; 7 Herbert Wertheim School of Public Health and Human Longevity Science, University of California, San Diego, La Jolla; 8 California Alzheimer’s Disease Center, University of California, San Francisco at Fresno; 9 Department of Neurology, Keck School of Medicine, University of Southern California, Los Angeles; 10 Department of Family Medicine, Keck School of Medicine, University of Southern California, Los Angeles

## Abstract

**Importance:**

Although the barriers to dementia care in primary care are well characterized, primary care practitioner (PCP) perspectives could be used to support the design of values-aligned dementia care pathways that strengthen the role of primary care.

**Objective:**

To describe PCP perspectives on their role in dementia diagnosis and care.

**Design, Setting, and Participation:**

In this qualitative study, interviews were conducted with 39 PCPs (medical doctors, nurse practitioners, and doctors of osteopathic medicine) in California between March 2020 and November 2022. Results were analyzed using thematic analysis.

**Main Outcomes and Measures:**

Overarching themes associated with PCP roles in dementia care.

**Results:**

Interviews were conducted with 39 PCPs (25 [64.1%] were female; 16 [41%] were Asian). The majority (36 PCPs [92.3%]) reported that more than half of their patients were insured via MediCal, the California Medicaid program serving low-income individuals. Six themes were identified that convey PCPs’ perspectives on their role in dementia care. These themes focused on (1) their role as first point of contact and in the diagnostic workup; (2) the importance of long-term, trusting relationships with patients; (3) the value of understanding patients’ life contexts; (4) their work to involve and educate families; (5) their activities around coordinating dementia care; and (6) how the care they want to provide may be limited by systems-level constraints.

**Conclusions and Relevance:**

In this qualitative study of PCP perspectives on their role in dementia care, there was alignment between PCP perspectives about the core values of primary care and their work diagnosing and providing care for people living with dementia. The study also identified a mismatch between these values and the health systems infrastructure for dementia care in their practice environment.

## Key Points

**Question:**

What are primary care practitioner (PCP) perspectives on their role in dementia diagnosis and care?

**Findings:**

In this qualitative interview study with 39 PCPs from across California, 6 themes associated with their perspectives on their role in dementia care were identified. These themes focused on their role in the diagnostic workup; the importance of long-term, trusting relationships with patients; the value of understanding patients’ life contexts; their work to educate families; their activities around coordinating dementia care; and the systems-level constraints they face.

**Meaning:**

These findings suggest PCPs believe they have an important role in providing dementia care that is not always facilitated by the health care systems in which they work.

## Teaser

This qualitative study evaluates primary care practitioner perspectives on their role in dementia diagnosis and care.

## Introduction

When it comes to dementia specifically, we’re there from the beginning to the end, or at least we can be. The thing that appeals to me about primary care is I have the training to deliver babies. I can be there when somebody takes their first breath as a human being. And then I have training to take care of somebody at the end of their life, too, when they take their last breath. And for every breath in between, we’re here. So, I think when it comes to people’s family systems and the life course that someone takes, we are uniquely positioned to take care of people with dementia, to be the medical provider.

Primary Care Practitioner 16

As the population ages, the number of people living with dementia will grow, as will primary care’s involvement in their care. In the US, primary care practitioners (PCPs) are often the first to recognize a patient has cognitive impairment. PCPs make most initial dementia diagnoses, are central to postdiagnostic care, and have a growing role in the dementia care workforce.^1,2^ Domestic and global policy recommendations advocate greater involvement of primary care in dementia care to address the world’s rapidly aging populations and the lack of available specialists.^3,4,5,6^ Primary care’s core values as a medical discipline are to improve population health, emphasize disease prevention and health promotion, build relationships with patients over time, account for patients’ family and community contexts, and coordinate care.^7^ Primary care is predominant in serving low income and rural communities in the US and globally because poverty and geography bar access to specialty care. Yet, as central as primary care is to patient health, it is severely underfunded in the US health care system. Moreover, how PCPs work is associated with the productivity and access standards set by the health systems they work in. These pressures can create enduring conflicts with the core values of primary care and limit opportunities for practice improvement. Due to these constraints, PCPs have reported difficulties meeting the needs of patients with complex chronic conditions such as dementia.^8,9,10,11,12,13,14,15,16^

A 2020 Alzheimer Association survey^2^ found that 82% of PCPs believe they are on the frontlines of dementia care, and 53% fielded questions related to dementia every few days. One recent study^1^ found that 64% of older adults who receive a dementia diagnosis in the US received their diagnosis from a PCP. PCPs are expected to play an increasingly important role in early detection, diagnosis, and ongoing care management for people with dementia as the population ages and new treatments emerge.^2^ However, many studies have identified endemic barriers to dementia care in primary care. Systemic issues such as lack of time, resources, and education are common themes. PCPs report lack of confidence and training to diagnose and manage dementia.^8^ PCPs also identify difficulties accessing and communicating with specialists, connecting with services, and managing patient and family preferences.^9,10,11,12,17,18,19,20,21,22,23,24^

Although barriers to dementia care in primary care are well characterized, we need a better understanding of PCPs’ perspectives on and values about the role of primary care in dementia. This study addresses this knowledge gap with the goal of identifying strategies to better support PCPs’ work in dementia. A multidisciplinary family medicine reform task force found that family medicine’s impact on public well-being is dependent on its ability to define its core values and implement health care reforms that ensure that care delivery is aligned with these values.^25^ Understanding PCPs’ values is thus critical to prepare, leverage, and support the primary care workforce needed to care for the growing population with dementia.

## Methods

### Design and Data Collection

This analysis was part of a larger qualitative study on PCP practices and attitudes regarding dementia diagnosis and care. We interviewed PCPs (medical doctors, nurse practitioners, and doctors of osteopathic medicine) in California between March 2020 and November 2022. Participants were recruited using purposive sampling. We sent emails to primary care colleagues and those engaged in research with neurology collaborators and asked them to connect us with PCPs in their practice networks. We oversampled practitioners who work in safety net settings that serve a high proportion of patients from racial and ethnic minority groups and those with low socioeconomic status. Two researchers, a PhD-trained medical anthropologist and a nurse (A.B.S. and C.A.), obtained oral consent and interviewed PCPs over Zoom. A multidisciplinary team developed the interview guide (eAppendix in Supplement 1) with the following domains: (1) professional background; (2) approach to dementia diagnosis and care; (3) experiences doing dementia diagnosis and care in populations who have increased vulnerability due to chronic comorbid conditions and historical, social, and/or structural inequalities; (4) PCP perspectives about their role in dementia; and (5) dementia-related training. Interviews lasted approximately 1 hour. PCPs self-reported demographic information in a survey; we collected these data to ensure equitable recruitment of practitioners from diverse backgrounds as well as to inform potential analyses of experiences among diverse practitioners. These data are not included in the present analysis. In this study we focused on PCP perspectives on their role in dementia care that emerged throughout the interview. This study was approved by the University of California, San Francisco’s institutional review board and follows the Standards for Reporting Qualitative Research (SRQR) guideline.^26^

### Data Analysis

Three researchers (A.B.S., M.M., A.H.) familiarized themselves with the data by reviewing transcripts. They developed a codebook according to deductive codes identified from study goals and review of the literature and then conducted deductive and inductive coding of 3 interviews together in ATLAS.ti version 22.2.1 (Scientific Software Development) to establish agreement in coding approach and code definitions. M.M. and A.H. divided the remaining transcripts and independently coded all data, double coding 7 transcripts. A.B.S. reviewed all coded data by examining transcripts, extracting quote reports, and reviewing reports for concordance with code definitions. When new concepts emerged, coders proposed additional inductive codes and reviewed transcripts to apply new codes. The team met weekly to review coding and resolve discrepancies via consensus. An in-depth memo was created to summarize each interview. All codes, quotes, and memos were examined to identify key themes and exemplary quotations.

## Results

We interviewed 39 PCPs across California (see Table 1 for demographics and practice characteristics). Twenty-five (64.1%) were female, 16 (41%) were Asian, 14 (35.8%) were White, 3 (7.6%) were Black or African American, 3 (7.6%) were Hispanic or Latino, 1 (2.5%) was Native Hawaiian or Pacific Islander, 1 (2.5%) was East Indian, and 1 (2.5%) was Middle Eastern. The majority (36 PCPs [92.3%]) reported that more than half of their patients were insured via MediCal, the California Medicaid program serving low-income individuals. We identified 6 themes regarding PCP perspectives on their role in dementia care. Findings demonstrated alignment between primary care core values and PCP perspectives on their role in dementia care (Table 2). Additional quotes are available in Table 3.

**Table 1.** Participant Characteristics

Characteristic	Participants, No. (%) (N = 39)
Sex	
Female	25 (64.1)
Male	14 (35.8)
Race and ethnicity	
Asian	16 (41)
Black or African American	3 (7.6)
East Indian	1 (2.5)
Hispanic/Latino	3 (7.6)
Middle Eastern	1 (2.5)
Native Hawaiian or Pacific Islander	1 (2.5)
White (non-Hispanic/Latino)	14 (35.8)
Specialty	
Medical doctor	30 (76.9)
Nurse practitioner	6 (15.3)
Doctor of osteopathic medicine	3 (7.6)
Years in practice	
<1	1 (2.5)
1-5	18 (46.1)
6-10	9 (23.0)
11-15	3 (7.6)
≥16	8 (20.5)
Prior training in dementia^a^	
Never received any formal training in this area	18 (46.1)
CME programs	15 (38.4)
Medical school, residency, or fellowship	5 (12.8)
Professional education through community groups such as Alzheimer Association	4 (10.2)
Working in a geriatric clinic	2 (5.1)
National conferences	2 (5.1)
Care for high percentage of older adults	1 (2.5)
Practice setting^b^	
Family medicine	7 (17.9)
Internal medicine	24 (61.5)
Geriatrics	6 (15.3)
Primary care or prevention medicine	1 (2.5)
Street medicine for those in emergency shelter situations	1 (2.5)
% Patients insured through MediCal	
>25	1 (25)
25-49	2 (5.1)
50-75	12 (30.7)
<75	24 (61.5)
Geographic region	
Southern California	20 (51.2)
SF Bay Area	16 (41)
Central Valley	3 (7.6)

Abbreviations: CME, continued medical education; SF, San Francisco.

^a^ Participants identified more than 1 training opportunity.

^b^ Participants identified more than 1 practice setting.

**Table 2.** Alignment of Primary Care Core Values With Dementia Care

Primary care core value	Alignment with dementia care
First point of contact	Identifying a cognitive problem
	Role in parts of dementia evaluation and treatment (cognitive testing, laboratory testing, imaging referrals, medications, and ongoing care)
	Ambivalent about ability to diagnose
Trusting, longitudinal relationships with patients	Enables communication about dementia
	Safe space during challenging diagnosis
Holistic view of patient contexts	Awareness of patients’ social, structural, and community circumstances
Care coordination	Navigating scope of practice with specialty neurologists
	Most refer for diagnosis
Health promotion	Responsibility to educate patients and families
Prevention	Dementia-specific prevention not discussed in interviews

**Table 3.** PCP Perspectives on Their Role in Dementia Care

Theme	Additional exemplary quotations (participant)
Role as first point of contact in detection and diagnostic workup	“I think as the first point of contact for family members, we should certainly be aware of how to approach the evaluations of dementia and, I think initial screening labs. I generally think, we should be aware of when we need to refer out, so when we’re uncertain or when we think we need to have a specialist weigh in.” (PCP08)
	“[Our role] is essential because we’re probably who has the most contact with patients…and I think noticing those changes over time is something that’s important for us to look out for and recognize. Also, I think having those more difficult discussions if we do have a good relationship with the patients makes sense instead of, you know, maybe even a neurologist for the first time being like, you know, I’m worried you can’t drive anymore.”(PCP26)
Longitudinal, trusting relationships	“Definitely in my experience with my patients, we’re the ones that they trust….They trust me, and that therapeutic relationship goes a long way.” (PCP17)
	“The workup and treatment should be done by the primary care provider because you already have that relationship with that patient. When you’re 70 or 80, it’s not like just go down the hall. You’re telling someone you need to find transportation; your children need to take the day off; you need to go to a whole new specialist that you’ve never met before and might not speak your language. That is a really tough sell.” (PCP09)
Holistic view of patients’ social and structural contexts	“Wanting to really build a long-term relationship with my patients. Getting to know them over time. Knowing every aspect of their lives. I think only primary care provides that luxury while being able to make sure that, you know, not only am I addressing medical needs, but also making sure that I’m addressing all the social determinants that are faced within this patient population itself.” (PCP 27)
	“To think about the patient in the broader context of their lives as opposed to the way a surgeon would look at, an orthopedist would look at a knee…a more holistic view of the patient and what’s reasonable given their environment.” (PCP07)
Responsible for involving families and providing education	“I think we try to prepare caregivers, family members, and also the patient for what may come....I generally like to make sure that the family members understand what they're getting into if they're going to be the caregiver and support them as well....I feel like the family member is my patient as well.” (PCP37)
	“Being that interface, almost like a translator…being able to really bridge the medical side of it all to the family on how to better take care of the patient.” (PCP32)
Coordinating care and scope of practice with specialists	**“**Even if they’re seeing a specialist, they can call the primary and say, ‘I’m having this problem,’ and then the primary can act as the go-between to the specialist. Because the specialist, they don’t have that bond with patients. They really don’t. They get less time and only need to focus on 1 problem. They don’t know about what’s going on at home, caregiver burnout. They don’t know about all that stuff.” (PCP34)
	“Where it’s a little bit challenging is when they’re slightly atypical or there’s any concern for something other than, say, Alzheimer’s and/or vascular dementia, that would be something that I would want to have a specialist weigh in to confirm.” (PCP28)
Mismatch between dementia care values and practical realities	“I would love to be fully equipped to care for my patients, including my geriatric patients. I wish I had some more geriatric training…I think I’m so limited by time. In an ideal world…[t]he system would allow for me to market a patient as someone who needed extra time, and I could give them a 40-minute appointment or an hour appointment instead and not get dinged for it in terms of my productivity….Instead, I’m using all of my own time to advance care for these patients. So, I think those are things that would help that philosophical thing become a reality, but I think until those things change, primary care physicians or I will continue to refer because I don’t have the time to do the assessment….In an ideal world, philosophically it should be the primary care provider with a consultation from a specialist, intermittent consultations. But I think the system would have to change to allow us to do that.” (PCP13)
	“We’re an overwhelmed society. We don’t really care. And that’s the health care system. We’ve got a lot going on. Do we follow up, if they miss their appointments, do we follow up with them? Do we assume that they don’t want to go to that appointment or do the labs? I hate to say it, but I think that that could be a component [in missed diagnosis].” (PCP34)

Abbreviation: PCP, primary care practitioner.

### Theme 1: Role as First Point of Contact

PCPs generally felt that they were the first people in the health care system to identify patients’ cognitive problems, whether by noticing something was wrong, such as a missed appointment or problems with medications, or when a family member or patient confided in them. They also felt they should be responsible for parts of the dementia evaluation and treatment, such as conducting a cognitive assessment, laboratory workup, seeking to delay the worsening of symptoms, and providing medications.

We’re responsible for identifying people who have potential dementia and doing initial workup, so being able to do some sort of cognitive assessment tool whether it’s us or some other staff in my clinic, and then do a basic lab workup and know when or not to order neuroimaging. And then referral and the supportive services that people need. I think the primary care clinic should be able to take on some of that whether it’s through social work, behavioral health integration, or complex care management.

PCP10

PCPs also understood the challenges patients with suspected dementia face navigating the health care system. However, the majority felt they needed confirmation of diagnosis from a specialist following a workup, even when they felt confident in their own diagnosis. Most expressed a desire to be more confident giving a diagnosis but felt that would require more training and time. PCPs overall reported minimal training in dementia (Table 1). Those who did have formal training reported exposure to content in medical school, residency, and through continued medical education courses, as well as using evidence-based resource support for point-of-care clinical decisions such as UpToDate.

### Theme 2: Trusting Relationships With Patients

Participants felt that PCPs were uniquely positioned to provide dementia care because of their trusting relationships with patients. They felt that the trust built through ongoing relationships enabled strong communication and helped decrease anxiety for patients in the face of a confusing and scary diagnosis. Many considered long-term relationships with their patients to be a privilege they valued highly.

I think trust is a big thing…dementia is a scary thing for patients. Having that core of trust in the relationship for established patients and being able to as a provider guide patients and caregivers through that…that’s golden….To be able to leverage that in the care of patients with dementia…[i]t’s a privilege…a real service that you can provide your patients.

PCP22

### Theme 3: Holistic View of Patients’ Life Contexts

Many PCPs described dementia care as part of their broader responsibility and capability to care for the whole person. Caring for the whole person, according to many participants, included maintaining the longitudinal relationships discussed previously and providing comprehensive care, including prevention, education, and psychosocial support. PCPs felt they saw patients’ lives in context, which not only provided them with the knowledge needed to detect memory changes—for example, noticing when a previously punctual patient missed appointments—but also made them established and reliable sources of support for patients and their families. These factors made PCPs feel uniquely positioned to understand dementia within the broader context of patient’s lives. Their awareness of family dynamics and how patients are connected to their communities enabled holistic dementia care that considers crucial contextual factors such as caregiver support and social determinants of health.

So much of the impact on a patient’s care is not going to be when I see them for 15 minutes. It’s going to be what’s going on in the outside world. That’s what I try and really focus on, is those connections of who’s important in your life? What are your goals for your life outside of this clinic room? And how can I be supportive in connecting you into those kinds of activities or care that you need in your home, in your community…those are the connections that I’m really trying to make after I’ve done a [dementia] assessment and make sure that I understand as much as I can about what’s going on outside of the clinic room.

PCP11

### Theme 4: Involving Families and Providing Education

Many PCPs discussed their role in involving family members when caring for people with dementia. When involving families, PCPs felt they had a role as educators, particularly in educating patients and their families about disease progression and helping them prepare for the future, such as engaging in advance care planning. Many PCPs also identified their role in interpreting findings for patients and family members and counseling them about the disease.

In terms of geriatric care, my philosophy is to provide as much assistance to the family as to the patient because ultimately, the care of the patient falls on the family. And if the family’s not well cared for, the patient will suffer the consequences. I really need to approach each patient as a family unit versus just the patient…whether the family member lost their job and is not able to provide food. Or whether they aren’t able to provide transportation and that’s another hindrance for the patient coming to the appointments.

PCP39

### Theme 5: Coordination of Care and Scope of Practice With Specialists

Many PCPs responded to the question about the role of PCPs in dementia care by discussing primary care as a place where overall care gets organized, as well as by discussing their scope of practice, role boundaries, and coordination in relation to specialists. Most PCPs felt that involvement of neurologists was helpful, particularly in making a diagnosis, and especially for atypical dementias they rarely see. PCPs also reported referring to specialty care due to limited bandwidth and when they felt their patients would receive better care and resources. However, many believed they themselves were better suited to provide ongoing dementia care management. They contrasted the shortage of neurologists and the nature of one-time specialist visits with primary care’s greater accessibility, established bond with patients, and knowledge of patient contexts.

We play the role of the ongoing care…I see the neurologist as kind of either saying, yes, this is the right diagnosis or, no, you’re missing something. We’re the ones who see these patients longitudinally and handle a lot of the complications of dementia whether they’re medical or behavioral.

PCP24

### Theme 6: Mismatch Between Dementia Care Values and Practical Realities

Most participants shared the belief that dementia care was within their scope of practice; however, they identified disparities between what they wanted to do and their ability to fulfill this role. Nearly all PCPs expressed a desire to be better equipped to provide dementia care and described ways that systemic challenges broadly impacted their ability to provide the care they wanted to offer. Impediments included short appointment times, lack of reimbursement and funding, and lack of staff support and resources that constrained their comfort, morale, and ability to provide comprehensive dementia care. Some believed that systemic change, including making dementia care a priority and bolstering institutional support for primary care, would be needed to realize their values in dementia care.

Philosophically, I think who else is better suited to [provide dementia care]? And then at the same time, we definitely don’t have the resources, the culture societally, or the systems and structures in place with reimbursement and funding to do it well.

PCP16

## Discussion

This study illustrated the connections between primary care values and PCP perspectives on their role in dementia care. Despite participants’ perspectives that dementia care was well-suited to their scope of practice, PCPs identified lack of confidence in providing an actual cognitive diagnosis and a mismatch between their values and practice capabilities due to systemic challenges. PCPs identified systems-level barriers that often negated their values as clinicians, suggesting the need for major practice reform to build real change, such as longer appointment times and different reimbursement structures. Prevention is central to primary care, but our participants did not raise the issue of brain health and dementia prevention; prevention of worsening disease was primarily discussed in connection to medications. Furthermore, it is notable that nearly half of the PCPs in our study identified no formal training in dementia.

The philosophy, values, and characteristics of primary care have been explored in educational and professional literature.^27,28,29,30,31^ Prior work has looked at PCPs’ approaches to caring for people with dementia, including the importance of trust-based relationships in advance care planning^32^ and attitudes toward caring in dementia care.^33^ We built on this work by focusing on PCP values; more work is needed to understand how to bridge gaps between values and common barriers. Further work is also needed on patient preferences for primary care involvement in dementia diagnosis and care.

Studies on the barriers to dementia care in primary care are numerous.^8,10,13,14,34,35,36,37,38,39^ Prior research found that PCPs underutilized available tools, lacked confidence in diagnosing and managing dementia, and there was often a delay between symptom onset and diagnosis.^9,10,11,12,14,15,16,21,40,41,42,43^ Results from our study showed that PCPs saw dementia care as consistent with their values and responsibilities as clinicians. Our results also suggested the need for interventions that support and empower PCPs to actualize these values in practice.

Supporting PCPs to deliver high-quality dementia care that aligns with their professional values has the potential to strengthen global policy efforts to involve primary care in dementia care, thus addressing the challenges of increasing prevalence of dementia, lack of specialists, and limited funding.^44^ For example, although there is a body of literature spanning multiple decades developed to guide PCPs on how to diagnose and manage dementia,^45,46,47,48,49,50,51,52^ PCPs we interviewed reported limited training in dementia. One approach to addressing this training gap is the Alzheimer and Dementia Care Extension for Community Healthcare Outcomes program (AD-ECHO).^53,54^ AD-ECHO involves a hub-and-spoke model of education and consultation, where PCPs engage in brief didactic sessions and present a case from their practice to dementia experts for discussion. This type of program can remedy training gaps and can also enable PCPs to gain access to specialty knowledge while keeping their patients within primary care.

Another strategy to support dementia care within primary care is a task-shifting approach that provides specialized dementia-focused case management or care navigation for people with dementia and their caregivers.^55,56,57,58,59,60^ In randomized trials, some task-shifting interventions have demonstrated improvement in dementia care quality, caregiver well-being, and patient outcomes.^56,57,59,60^ A systematic review^61^ of evidence-based interventions targeting primary care found that PCP–case management partnership models were the most promising in impacting neuropsychiatric symptoms, caregiver burden, and health care costs. These approaches may ultimately improve PCP experience by relieving systems-level constraints and enabling PCPs to be part of a team that provides more comprehensive dementia care. Furthermore, the Centers for Medicare and Medicaid Services recently announced the new US-based Guiding an Improved Dementia Experience Model that focuses on dementia care management through the provision of care coordination, care management, caregiver education and support, and respite services.^62^ This alternative payment for the delivery of these services may enable PCPs to better support their patients in settings where such programs exist. However, more work is needed to understand how training, task-shifting, additional roles in the health care system, and new policies may change practitioner experience and alignment with PCP values of care.

Our results also suggest that the primary care mission and values are consistent with a comprehensive, whole-person approach that involves both patients and caregivers, such as the Six Domains of Health framework developed by Sadak and Borson.^63^ This kind of framework may help PCPs set priorities and identify the support they need to provide high-quality, value-aligned, and comprehensive care for their patients with dementia. This framework suggests the use of simple tools to inform assessment and care planning for people with dementia and their caregivers, and designates specific care team roles that focus on both individual health priorities and social care. This approach supports PCPs’ desires to holistically understand patients’ needs and social settings and to involve family in dementia care. Our results illustrated that this type of model could enable PCPs to better align the reality of their dementia care practice with their values.

The quotation from PCP16 in this article’s introduction highlighted the professional context of this study. Our results showed that most PCPs in our study wanted to provide the social and emotional care required by people with dementia and their caregivers. Our study also raised the question of what needs to change for PCPs to realize these values in practice.

We believe that changes in 3 domains may help bring dementia care into better alignment with PCP values. First, PCPs’ role in the health care system needs to be elevated and better valued in recognition of the important part they play over time in their patients’ lives and in communities. Second, more research is needed on how to build and better study the PCP experience of collaborative models and streamlined training and communication that may facilitate better access to specialty care or specialty consult to complement PCPs’ work.^64^ Third, PCPs need better payment models for dementia care and to be given more time for dementia-specific visits.^65^ Reimbursement and time would help PCPs actualize their mission-driven values in practice with patients facing this complex diagnosis, as well as to have the time to involve family members, an important aspect of dementia care. Focusing on these policy interventions and building better infrastructures of support can help better connect the work PCPs do with their values about dementia care.

### Limitations

This study has several limitations. Selection bias may have identified PCPs who are more committed to dementia care, whether due to interest or participation in a related study introducing new dementia diagnostic tools. Also, we recruited PCPs working primarily in safety net settings, which does not represent the heterogeneity of primary care perspectives. Furthermore, half of our participants were less than 5 years from residency. Additionally, more work is needed to understand patient perspectives on the role of primary care in dementia care.

## Conclusions

In this qualitative study of PCP perspectives on their role in dementia care, we found alignment between the core values of primary care and what they consider important in diagnosing and caring for people living with dementia. We also identified opportunities to develop health systems infrastructure that better aligns with PCP values and supports dementia care in primary care. Understanding and addressing PCP values when designing new organizational strategies and implementing new tools and guidelines thus may represent an opportunity to improve care and care processes, even in settings where time and resources are constrained. These changes are critical to better support primary care work in dementia.

## Notes

Supplement 1. **eAppendix.** Primary Care Practitioner In-Depth Interview

Supplement 2.
Data Sharing Statement
