# Error in Title

**DOI:** 10.1001/jamanetworkopen.2023.42418

**Published:** 2023-10-10

In the Original Investigation titled “Primary Care Practitioner Perspectives on the Role of Primary Care in Dementia Diagnosis and Care,”^1^ published September 28, 2023, “practitioner” was misspelled in the title. This article has been corrected.^1^
