# Supplementary material for: Primary Care Practitioner Perspectives on the Role of Primary Care in Dementia Diagnosis and Care
Source: JAMA Netw Open. 2023 Sep 28;6(9):e2336030. doi: 10.1001/jamanetworkopen.2023.36030 (PMC10539983; doi:10.1001/jamanetworkopen.2023.36030)
Supplement: Supplement 1. — eAppendix. Primary Care Practitioner In-Depth Interview [file jamanetwopen-e2336030-s001.pdf]

Supplemental Online Content

Sideman AB, Ma M, Hernandez de Jesus A, et al. Primary care practitioner perspectives on the role of primary care in dementia diagnosis and care. *JAMA Netw Open*. 2023;6(9):e2336030. doi:10.1001/jamanetworkopen.2023.36030

eAppendix. Primary Care Practitioner In-Depth Interview

This supplemental material has been provided by the authors to give readers additional information about their work.

© 2023 Sideman AB et al. *JAMA Network Open.*

Primary Care Practitioner In-Depth Interview

Background

- Could you please describe your practice setting and the patient population you work with?

Diagnostic process and clinic flow

- Can you describe what you would typically do with a patient who you suspect has dementia? Walk me through the process.
    - Prompts,
        - *What are red flags? How do you decide whether to evaluate a patient for dementia?*
        - *How do you raise the topic of dementia or memory issues with your patients if you are concerned?*
        - *What would your first step be?*
        - *What does your workup involve?*
            - *Labs?*
            - *Imaging?*
            - *Cognitive testing?*
        - *How do you decide what to do next?*
        - *Who is involved in the process? What are their roles?*
- Do you ever give a diagnosis of dementia or Alzheimer's disease? If not, why?
- How does your care of the patient change once they've received a dementia diagnosis?
- What types of barriers or roadblocks do you experience when caring for a patient with suspected dementia? With diagnosed dementia?
- What do you think works well in your diagnosis or care of patients with dementia?

Comorbidities

- How do other comorbidities impact your workup and care of patients with dementia?
- How does a dementia diagnosis impact ongoing care of a patient's comorbidities?
- Which comorbidity is the hardest to co-manage with dementia and why?
- What strategies do you use when you have a patient with dementia and other chronic comorbidities?

Underserved/underrepresented patients and those with social risks

- What are the challenges when doing dementia assessment and care with patients from underserved and underrepresented populations or those facing historical, social, and/or structural inequalities?
- What are the opportunities or strengths in your practice when doing dementia assessment and care with patients from underserved and underrepresented populations or those facing historical, social, and/or structural inequalities?

Co-management

© 2023 Sideman AB et al. *JAMA Network Open.*

- Can you describe your experience working with neurologists when you refer or co-manage a patient? What works well, what is challenging?
- How do you communicate with the neurologist?
- What kind of information do you receive from the neurologist?
    - What do you do with this information?

Caregiver support

- When a patient has a caregiver or informant involved, how, if at all, do you identify and address the caregiver's needs?
- What do you do when a patient has no caregiver?

Training/Needs

- Describe any specific training or experience you have had with dementia assessment, diagnosis, or care.
- What additional training or experience do you think you need to feel more comfortable doing dementia assessment, diagnosis, and care?

PCP Background

- Could you tell me about what led you to become a primary care provider?
- Have you had any personal experiences with dementia?
    - If yes, how, if in any way, has this shaped the way you think about dementia care in your practice?

PCP Values

- What are your values or beliefs about the role of primary care providers in dementia assessment, diagnosis, and care?

© 2023 Sideman AB et al. *JAMA Network Open.*
