# Supplementary material for: Primary Care Practitioner Perspectives on the Role of Primary Care in Dementia Diagnosis and Care
Source: JAMA Netw Open. 2023 Sep 28;6(9):e2336030. doi: 10.1001/jamanetworkopen.2023.36030 (PMC10539983; doi:10.1001/jamanetworkopen.2023.36030)
Supplement: Supplement 2. — Data Sharing Statement [file jamanetwopen-e2336030-s002.pdf]

Data Sharing Statement

Sideman. Primary Care Provider Perspectives on the Role of Primary Care in Dementia Diagnosis and Care. *JAMA Netw Open*. Published September 27, 2023. doi:10.1001/jamanetworkopen.2023.36030

Data
Data available: No
